# Radiation-induced brachial plexopathy in patients with nasopharyngeal carcinoma: a retrospective study

**DOI:** 10.18632/oncotarget.7748

**Published:** 2016-02-26

**Authors:** Zhaoxi Cai, Yi Li, Zhen Hu, Ruying Fu, Xiaoming Rong, Rong Wu, Jinping Cheng, Xiaolong Huang, Jinjun Luo, Yamei Tang

**Affiliations:** ^1^ Department of Neurology, Sun Yat-Sen Memorial Hospital, Sun Yat-Sen University, Guangzhou, Guangdong Province, China; ^2^ Key Laboratory of Malignant Tumor Gene Regulation and Target Therapy of Guangdong Higher Education Institutes, Sun Yat-Sen University, Guangzhou, Guangdong Province, China; ^3^ Department of Radiology, Sun Yat-Sen Memorial Hospital, Sun Yat-Sen University, Guangzhou, Guangdong Province, China; ^4^ Department of Neurosurgery, Sun Yat-Sen Memorial Hospital, Sun Yat-Sen University, Guangzhou, Guangdong Province, China; ^5^ Departments of Neurology and Pharmacology, Temple University School of Medicine, Philadelphia, PA, USA; ^6^ Guangdong Province Key Laboratory of Brain Function and Disease, Zhongshan School of Medicine, Sun Yat-sen University, Guangzhou, Guangdong Province, China

**Keywords:** nasopharyngeal carcinoma, brachial plexopathy, radiotherapy, MRI, electromyography

## Abstract

Radiation-induced brachial plexopathy (RIBP) is one of the late complications in nasopharyngeal carcinoma (NPC) patients who received radiotherapy. We conducted a retrospective study to investigate its clinical characteristics and risk factors. Thirty-onepatients with RIBP after radiotherapy for NPC were enrolled. Clinical manifestations of RIBP, electrophysiologic data, magnetic resonance imaging (MRI), and the correlation between irradiation strategy and incidence of RIBP were evaluated. The mean latency at the onset of RIBP was 4.26 years. Of the symptoms, paraesthesia usually presented first (51.6%), followed by pain (22.6%) and weakness (22.6%). The major symptoms included paraesthesia (90.3%), pain (54.8%), weakness (48.4%), fasciculation (19.3%) and muscle atrophy (9.7%). Nerve conduction velocity (NCV) and electromyography (EMG) disclosed that pathological changes of brachial plexus involved predominantly in the upper and middle trunks in distribution. MRI of the brachial plexus showed hyper-intensity on T1, T2, post-contrast T1 and diffusion weighted whole body imaging with background body signal suppression (DWIBS) images in lower cervical nerves. Radiotherapy with Gross Tumor volume (GTVnd) and therapeutic dose (mean 66.8±2.8Gy) for patients with lower cervical lymph node metastasis was related to a significantly higher incidence of RIBP (*P*<0.001). Thus, RIBP is a severe and progressive complication of NPC after radiotherapy. The clinical symptoms are predominantly involved in upper and middle trunk of the brachial plexus in distribution. Lower cervical lymph node metastasis and corresponding radiotherapy might cause a significant increase of the RIBP incidence.

## INTRODUCTION

Nasopharyngeal carcinoma (NPC) is a common malignant tumor and highly prevalent in East Asia [1–3], especially in Southern China. Radiotherapy remains the primary treatment of the choice for NPC, which may adversely cause complications [4]. Implementation of three-dimensional conformal radiotherapy (3D-CRT) and intensity modulated radiation therapy (IMRT) permits a precise dose emitting, and significantly reduces complications of the radiotherapy [5–7]. Delayed onset of radiation-induced neural tissue damage may occur, including temporal lobe necrosis, cranial nerve injury, and cognitive impairment [8–10]. Although radiation-induced plexopathy has been documented in patients with neoplasms of the mediastinum, breast, lung and lymph nodes, there is paucity in literature on radiation-induced brachial plexopathy (RIBP) in patient with NPC after radiotherapy [11–18]. Additionally, relationships between the cervical lymph node infiltration, corresponding radiation planning and incidence of RIBP have never been discussed. In this study, patients with RIBP after radiotherapy for NPC were retrospectively analyzed in order to address the clinical characteristics, electrodiagnostic studies and magnetic resonance imaging (MRI) scan of RIBP, and to elucidate the risk factors of RIBP after radiation for NPC.

## RESULTS

### Individual characteristics

A total of 31 cases of RIBP patients and 62 arms of brachial plexus were studied. Age of the onset of RIBP ranged from 31 to 83 years, mean 52.95±11.35 years. Thirty of 31 patients had metastases to the unilateral or bilateral cervical lymph node, and 28 of 31 patients received chemotherapy (Table 1).

**Table 1:** Patient and tumor characteristics

Characterisitics	Number of patients (n=31)	No (%)
Sex		
Male	19	61
Female	12	39
N-category		
N0	1	3
N1	12	39
N2	10	32
N3	8	26
Chemotherapy history	28	90

### Clinical characteristics

The interval between radiotherapy and the appearance of the first symptom varied from 6 months to 12 years, with a median of 4.26±2.26 years. Of the 31 patients, sixteen patients (16/31, 51.6%) complained of paraesthesia as the first presenting symptom. Numbness and hypoesthesia in the fifth to sixth cervical vertebrae (C-5∼C-6) nerves dermatomes at shoulder and lateral border of the forearm were predominant (28/31, 90.3%). Thumb and index fingers were also involved, with initial sparing of the digitus minimus manus. The second common symptom was persistent or interval pain (17/31, 54.8%), presenting the form of soreness, tingling, sharp knifelike or electronic shock-like pain at the neck, shoulder and upper arm. The later onset of symptom of RIBP was weakness (15/31, 48.4%), involving shoulder abduction and elbow flexion, and indicating C-5∼C-6 myotomes involvement. Diminished or absent tendon reflexes in the upper extremities and amyotrophy (3/31, 9.7%) and muscle fasciculation (6/31, 19.3%) in the proximal muscles were shown in some patients. Plexus neuropathy was graded using a modified LENT-SOMA score (Table 2).

**Table 2:** Clinical Characteristics of RIBP

	Number of patients(n=31)	N%
First symptom in onset		
Paraesthesia	16	51.6
Pain	7	22.6
Weakness	7	22.6
Involuntary movements	1	3.2
Major clinical manifestations		
Paraesthesia	28	90.3
Pain	17	54.8
Weakness	15	48.4
Fasciculation	6	19.3
Amyotrophy	3	9.7
LENT-SOMA scale		
Grade 1	4	12.9
Grade 2	11	35.5
Grade 3	14	45.2
Grade 4	2	6.5

### EMG and NCV manifestation

**Figure 1:**
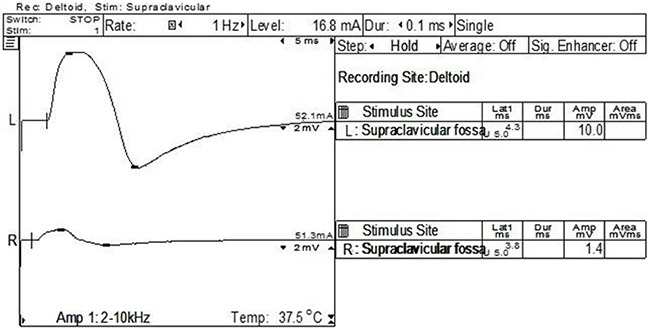
NCV of one case with right RIBP The right amplitude of MNC was much lower than that of the contralateral axillary nerve (1.4 mV at the right vs 10 mV at the left).

**Figure 2:**
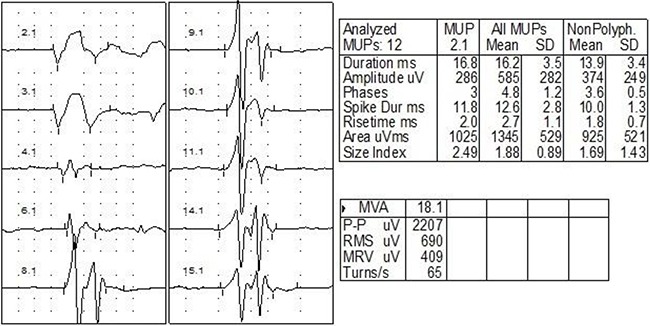
EMG of one case with right RIBP EMG showed prolonged duration and increased amplitude for the action potential of right deltoid (duration 16.2±3.5 ms; amplitude 585±282 μV).

**Figure 3:**
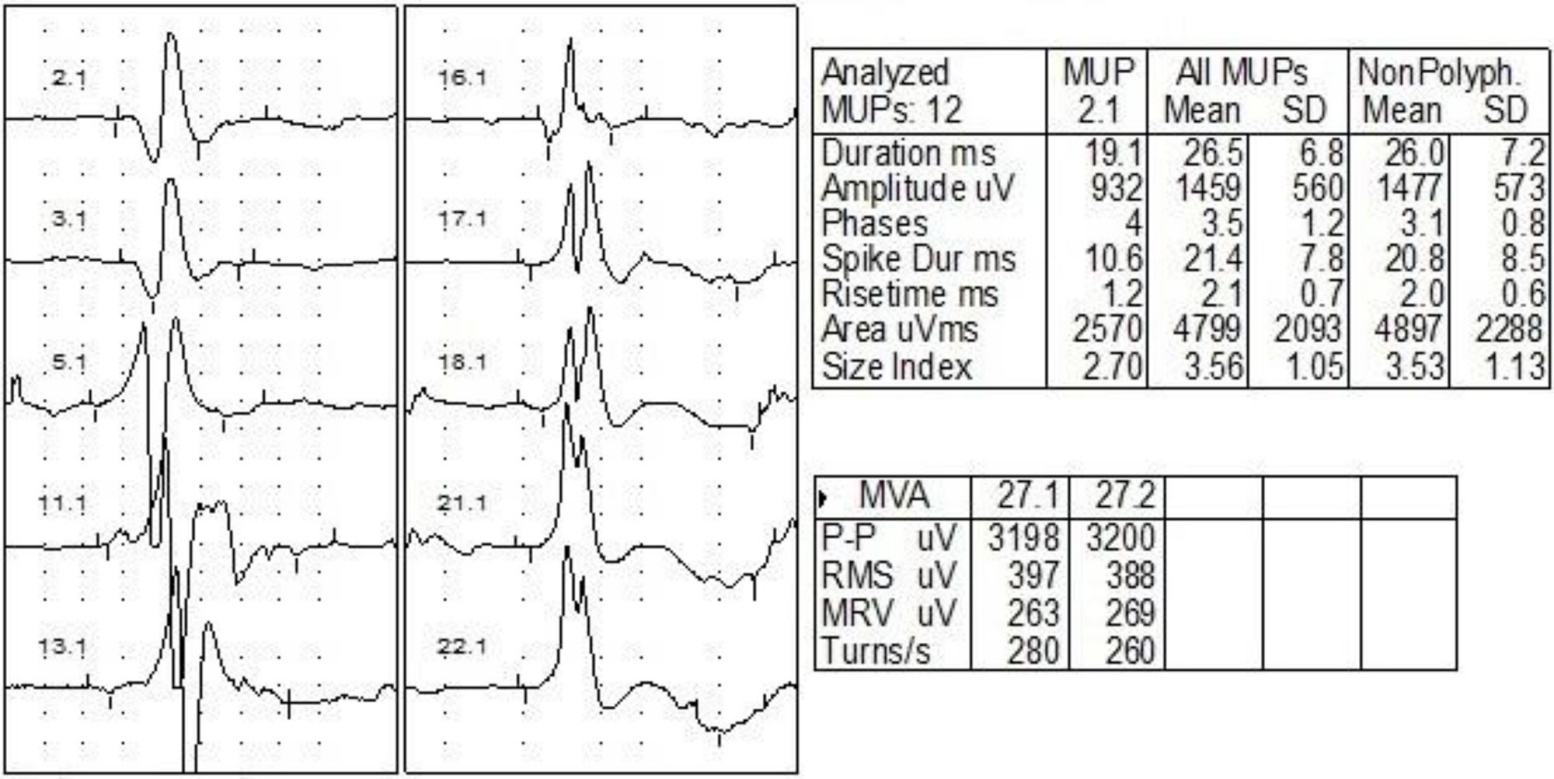
EMG of one case with bilateral RIBP EMG showed prolonged duration and increased amplitude for the action potential of biceps branchi (duration 26.5±6.8 ms; amplitude 1459±560 μV).

NCV and EMG confirmed an extensive brachial plexus lesion, which was mostly located in the upper and middle trunks. Involvement of lower trunk was relatively less. Decreased amplitude and velocity of sensory nerve conduction (SNC) and motor nerve conduction (MNC) were observed in axillary nerve (Figure 1) (31/31), musculocutaneous nerve (31/31), and the median nerve (13/31). Neurogenic damage of deltoid (31/31) (Figure 2) and musculus biceps brachii (31/31) (Figure 3) were observed. Besides, F wave abnormality and myokymic discharges, indexes of peripheral nerve injury, were also common in RIBP patients (Table 3).

**Table 3:** EMG and NCV of RIBP

	Number of patients (n=31)	No (%)
Localization of injury		
C-5∼C-7	31	100.0
C-8/T-1	13	41.9
Manifestation		
Abnormality of SNC	31	100.0
Abnormality of MNC	21	67.7
Longer F-wave	17	54.8
Myokymic discharges	26	83.9

### MRI manifestation

**Figure 4:**
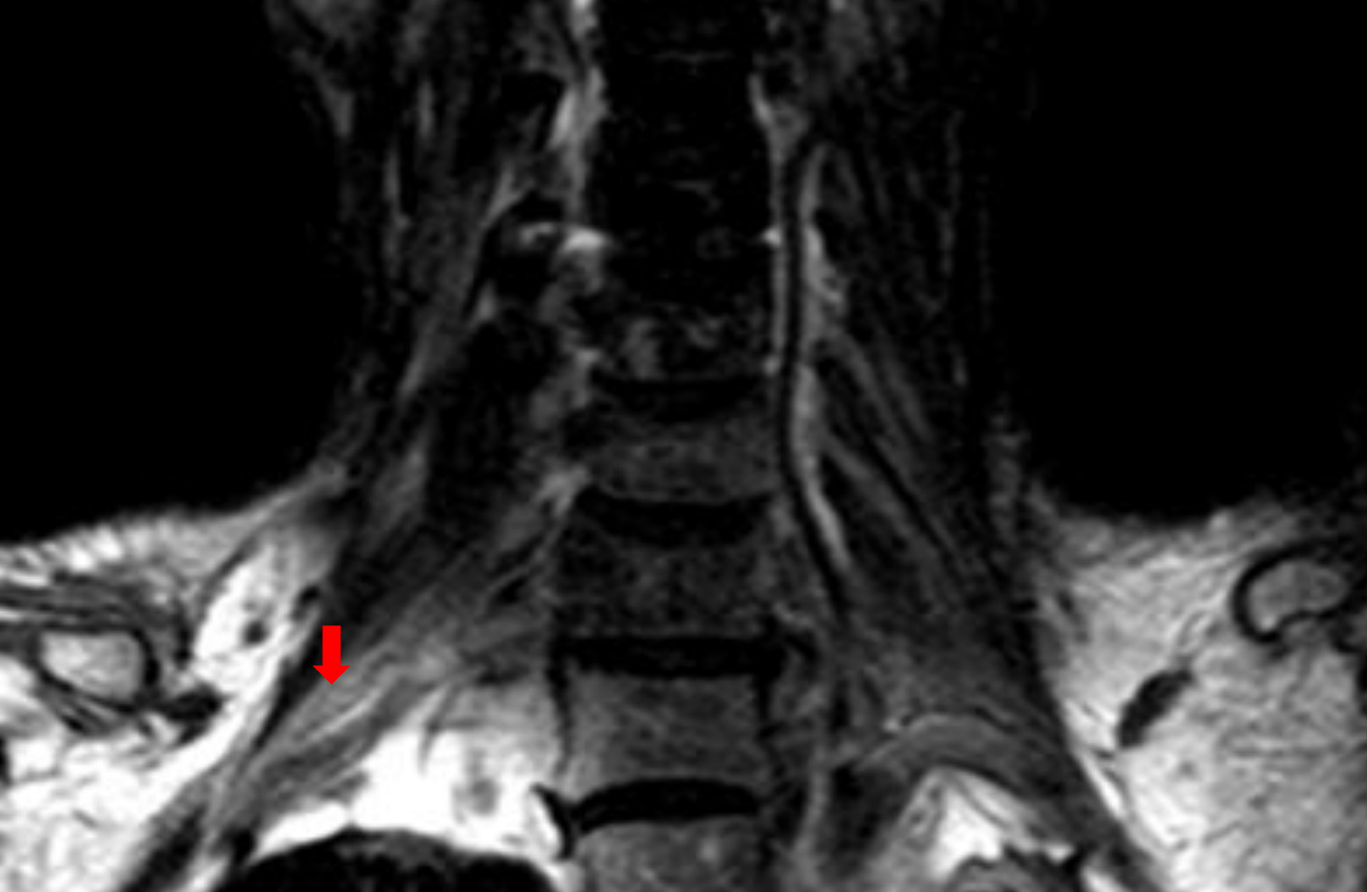
The T1-weighted coronal MRI scan of one case with unilateral RIBP The MR imaging showed high intensity within the left brachial plexus (red arrow), compared with the normal right brachial plexus.

**Figure 5:**
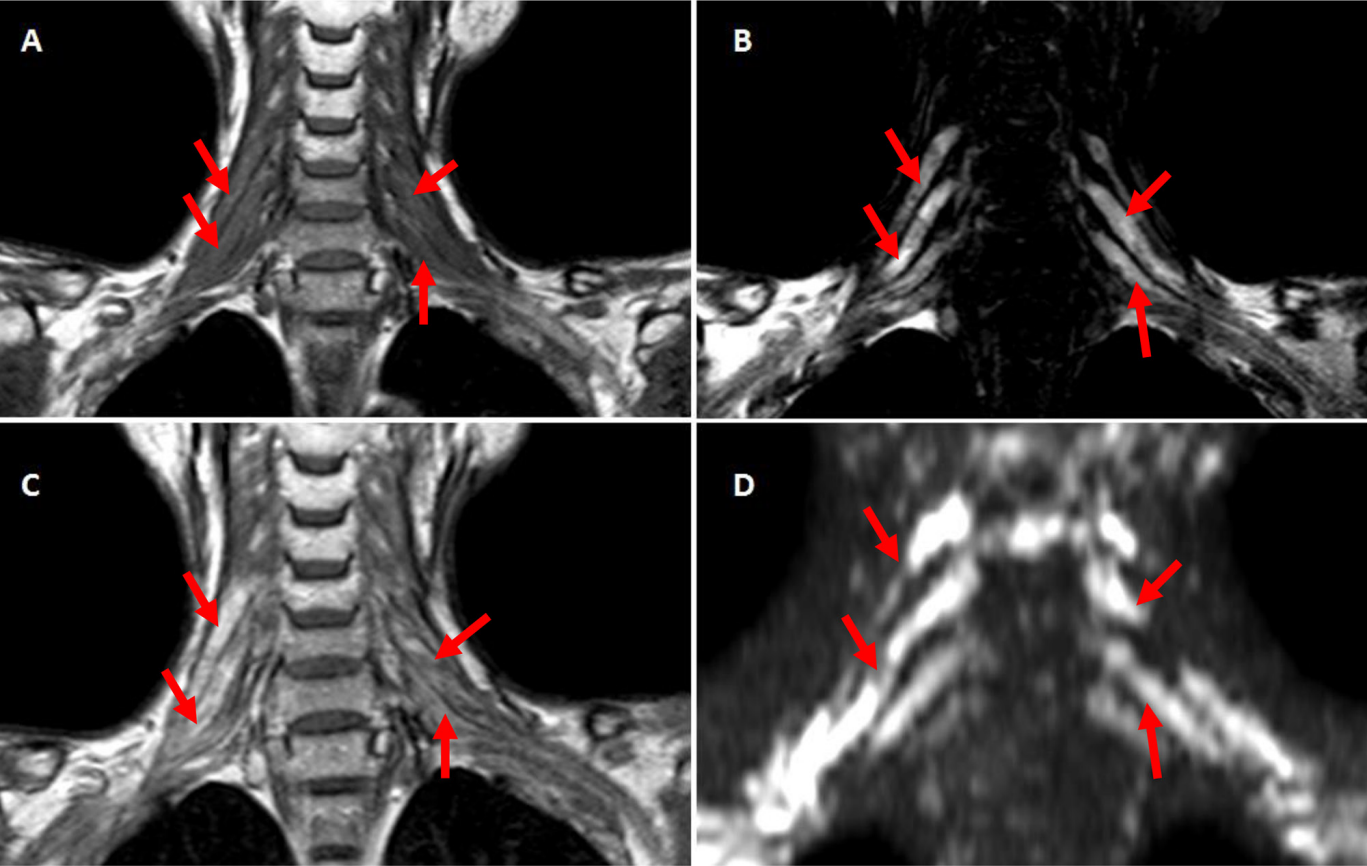
**A-D.** The coronal MRI scan of one case with bilateral RIBP T1-weighted coronal MRI scan showed bilateral brachial plexus were swollen with hyper-intensity. (A) C-5∼C-8 roots and corresponding trunks of brachial plexus showed prolonged T2 relaxation time at T2-weighted MR imaging (B), high intensity at Post-contrast T1-weighted imaging (C) and DWIBS (D) (indicated with red arrows).

MRI of brachial plexus excluded tumor relapse in all the patients. Short tau inversion recovery (STIR) showed asymmetrical polyradiculopathy identified by unilateral or bilateral swollen in cervical roots extending through to the trunks. These abnormalities are common in C-5∼C-8 levels but rare in the first thoracic vertebrae level (T-1). The injured nerves showed hyper-intensity on T1 and T2 weighted MRI images (Figure 4, Figure 5). The lesions of 8 patients showed enhancement at enhanced T1-weighted image (Figure 5C). Diffusion weighted MR imaging showed high intensity in the affected brachial plexus, indicating nerve edema (Figure 5D). Adjacent tissue surrounding the plexus was usually normal and intact.

### Relationship between radiotherapy and RIBP

Sixty-two Brachial plexus of 31 RIBP patients were divided into RIBP group (46 brachial plexus) and control group (16 brachial plexus). RIBP incidence showed significant differences among the radiotherapy categories (*χ*^2^=24.090, *P*=0.000<0.05, Table 4). Furthermore, multiple comparison among subgroups showed that radiotherapy to level IV or Vb metastasis caused a significant increase of RIBP as compared with the other two radiation categories (*P*=0.000<0.05, Table 4).

**Table 4:** Radiotherapy categories and brachial plexus neuropathy (*Chi*-Square test)

Radiation categories	Radiation fields		RIBP Group	Normal Group	Total
	GTVnd	CTVnd			
1	negative	II+III+Va	1	5	6
2	II, III or Va	II∼V	12	10	22
3	IV or Vb	II∼V+SCF*	33	1	34^a^^,^^b^

a *P*=0.000 vs radiation category 1;

b *P*=0.000 vs radiation category 2;

* SCF, supraclavicular fossa

## DISCUSSION

The brachial plexus innervates the sensory and motor function of upper limb. Because of its location, radiation-induced damage to the brachial plexus can occur in patients who have had radiation therapy for neoplasm of the mediastinum, breast, lung or lymph nodes [13, 15, 16, 18]. RIBP following radiotherapy of NPC is a latent but progressive disease, and is usually neglected by patients in the early stage. In this retrospective study, we found that as a severe complication of NPC after radiotherapy, RIBP mainly caused sensory and motor abnormalities in upper limbs. NCV, EMG and MRI are important examinations for diagnosis. Lower cervical lymph node metastasis and corresponding radiotherapy might cause a significant increase of the RIBP incidence.

In our study, paraesthesia was the most common symptom of RIBP, usually ignored by patients. Only would they seek medical attention when intolerable pain, weakness and myoatrophy occurred. Half patients in the study had been classified Grade 3 or 4 at the first visit for RIBP. The early symptoms of RIBP usually included numbness, hyperesthesia or pain on the neck, shoulder and lateral border of forearm. Thumb and index finger could also be involved, but the digitus minimus manus was relatively spared. Weakness and amyotrophy were the later symptoms of RIBP, usually involved shoulder abduction and arm flexors. The neurologic manifestation and signs in our patients were predominantly in upper and middle trunk distribution, which had been confirmed by electrodiagnostic studies. In patients of RIBP after breast cancer, lower trunk, e.g. C-8 or T-1, was commonly involved [21, 22], and pan-brachial plexus injury might occur in a prolonged clinical course [23]. This may be due to the differences of the radiation location between breast cancer and NPC. The area of axilla or infraclavicular fossa is commonly involved in the radiation for breast cancer, and the supraclavicular fossa is commonly involved in the radiation for NPC.

MRI is an important assistant examination for differential diagnosis of neoplasm invasion and RIBP [24–26]. Wouter et.al reported swelling of the plexus and hyper-intensity within the trunks on T2-weighted MR imaging in breast cancer [27], which is consistent with those in our study. Radiation-induced fibrosis of supraclavicular and axillary soft tissues was also described in a significant number (about 66.7%) of patients as a reduction in signal intensity of the adipose tissue in T1 and T2 weighted sequences [24, 26, 28, 29]. In contrast, the adjacent soft tissue surrounding the plexus in our patients was usually well preserved.

In this study, the statistical analysis indicated that the radiotherapy dosage to the neck dominated by N stage of cervical lymph node metastasis might be related to the development of RIBP. The cervical lymph node can be briefly divided into the upper cervical group (II, III and Va) and lower cervical group (IV and Vb). Neck node involvement by NPC is by orderly spread down the neck, and skipping metastasis is rarely seen [30]. For the incidence of RIBP, no significant difference was shown among N0, N1b, and N2 stages. However, in conditions that cervical metastasis spread to the lower neck (N3 stage), therapeutic radiation dose to the lower neck significantly increased the incidence of RIBP as compared with N0, N1b, and N2 stages. Although the factors affecting the risk and severity of radiation-induced nerve injury are not quite specific, several radiation-related factors have been identified, e.g. large total dose (>50 Gy to plexus, >60 Gy to cranial nerves), RT volume including a large proportion of nerve fibers, or heterogeneous high-dose distribution [31]. In conditions of lower neck metastasis, radiation to supraclavicular fossa was always proposed. Moreover, as compared with NPC patients of N0, N1b, and N2 stages, patients of N3 stage was applied with higher radiation dose (66.8±2.8Gy vs 53.6±2.3Gy) to the lower cervical lymph node, which might cause fibrosis of the surrounding tissue of brachial plexus.

As for the treatment of radiation-induced nerve injury, immunotherapy might be the first choice to date [32, 33]. Steroids were commonly applied to the RIBP patients in our study, and showed clinical relief (data not shown). Nonsteroidal anti-inflammatory drugs, e.g. immunoglobulin, would be applied to patients with contraindications to steroids. For the advanced stage of RIBP (LENT-SOMA scale greater than 3), treatment should focus on effective management of pain and rehabilitation for neuromuscular dysfunction. Pain and paraesthesia secondary to brachial plexus neuralgia induced by radiation are typical symptoms of neuropathic pain. First-line treatment for neuropathic pain includes tricyclic antidepressant (e.g. amitriptyline), antiepileptics (eg. carbamazepine, pregabalin) and opioids [34]. Although Vitamin B group and neurotrophic therapy, e.g. gangliosides, may improve the general outcome of patients with peripheral nerve injury or diseases, no considerable evidence indicates benefit for RIBP [35, 36]. Surgical intervention is unnecessary for treating RIBP in most cases, owing to a relative rarity of radiation-induced fibrosis surrounding the plexus. However, surgical exploration also allows the nerve to be released from fibrotic tissue. Omentoplasty, or periscapular amputation, an aggressive treatment, might be considered as a choice of treatment when RIBP has progressed to severe nerve injury and neurovascular involvement [37, 38].

## MATERIALS AND METHODS

### Patients

The charts of patients with a clinical diagnosis of NPC and finished a planned radiation using 3D-CRT or IMRT in Sun Yat-Sen Memorial Hospital and Sun Yat-Sen University Cancer Center, were retrospectively retrieved and collected between August 2008 and August 2013. RIBP was diagnosed according to a history of radiotherapy after NPC, clinical symptoms, neurological examination, electrophysiological examination and MRI. Exclusion criteria included the history of cancer relapse, metastases, multi-radiations, cervical lymph node dissection, previous history of brachial plexus neuropathy caused by trauma, infection, cervical spondylopathy, or allergic reaction. The following parameters were recorded: (1) demographics: age, sex, TNM staging of NPC, post-radiotherapy interval, radiation dose, radiotherapy techniques, and chemotherapy history; (2) clinical characteristics: first symptom at onset, major clinical manifestation of RIBP, cervical MRI and EMG. Plexopathy was graded using modified LENT-SOMA scale (late effects of normal tissue- subjective, objective, management, analytic).

### Radiation therapy technique

Radiotherapy (3D-CRT or IMRT) was performed based on N stage according to an institutional protocol at Cancer Center of Sun Yat-Sen University. N stage of the cervical lymph nodes was classified according to the 7th edition of the Union of International Cancer Control (UICC) and American Joint Committee on Cancer (AJCC) tumor, node, metastasis staging systems. Cervical node levels were divided into upper neck lymph drainage region (level II, III, and Va), and the lower neck lymph node drainage region (level IV, Vb and the supraclavicular regions) [19, 20]. The radiation dosage was conducted based on the N stage of cervical lymph node metastasis. Gross Tumor Volume of neck node (GTVnd) represented the metastasis-positive lymph node region with an average of 66.8±2.8Gy radiation dosage. Clinical Target Volume of neck node (CTVnd) presented the total area for radiation with prophylactic radiotherapy (53.6±2.3 Gy). In brief, radiotherapy of 3D-CRT/IMRT to the cervical lymph node was classified into 3 categories (Table 4). For stage N0 patients, GTVnd was conducted to II, III and Va regions. For patients with neoplasm metastasized to level II, III or Va, GTVnd was applied to the metastasis-positive lymph node region, and CTVnd was applied to ipsilateral level IV and Vb areas. Patients with infiltrated level IV or Vb region were conducted with GTVnd to level IV or Vb, and CTVnd to ipsilateral supraclavicular region.

### Statistical analysis

The incidence of RIBP with different radiation methods were compared and analyzed using 3×2 *Chi*-Square test. A two-tailed *P* values of <0.5 were considered significant. The SPSS for windows, version 18.0 was used for data processing.

## CONCLUSION

RIBP is a progressive disease in NPC patients after radiotherapy. The clinical symptoms are predominantly involved in upper and middle trunk of the brachial plexus in distribution. Lower cervical lymph node metastasis and corresponding radiotherapy might cause a significant increase of the RIBP incidence.

## References

[1] Zhang LF, Li YH, Xie SH, Ling W, Chen SH, Liu Q, Huang QH, Cao SM (2015). Incidence trend of nasopharyngeal carcinoma from 1987 to 2011 in Sihui County, Guangdong Province, South China: an age-period-cohort analysis. Chin J Cancer.

[2] Wei KR, Zheng RS, Zhang SW, Liang ZH, Ou ZX, Chen WQ (2014). Nasopharyngeal carcinoma incidence and mortality in China in 2010. Chin J Cancer.

[3] Shin HR, Masuyer E, Ferlay J, Curado MP (2010). Asian Contributors to CI. Cancer in Asia - Incidence rates based on data in cancer incidence in five continents IX (1998-2002). Asian Pac J Cancer Prev.

[4] Yee D, Hanson J, Lau H, Siever J, Gluck S (2006). Treatment of nasopharyngeal carcinoma in the modern era: analysis of outcomes and toxicity from a single center in a nonendemic area. Cancer J.

[5] Hsiung CY, Yorke ED, Chui CS, Hunt MA, Ling CC, Huang EY, Wang CJ, Chen HC, Yeh SA, Hsu HC, Amols HI (2002). Intensity-modulated radiotherapy versus conventional three-dimensional conformal radiotherapy for boost or salvage treatment of nasopharyngeal carcinoma. Int J Radiat Oncol Biol Phys.

[6] Lai SZ, Li WF, Chen L, Luo W, Chen YY, Liu LZ, Sun Y, Lin AH, Liu MZ, Ma J (2011). How does intensity-modulated radiotherapy versus conventional two-dimensional radiotherapy influence the treatment results in nasopharyngeal carcinoma patients?. Int J Radiat Oncol Biol Phys.

[7] Peponi E, Glanzmann C, Kunz G, Renner C, Tomuschat K, Studer G (2010). Simultaneous integrated boost intensity-modulated radiotherapy (SIBIMRT) in nasopharyngeal cancer. Strahlenther Onkol.

[8] Behin A, Delattre JY (2004). Complications of radiation therapy on the brain and spinal cord. Semin Neurol.

[9] Kong L, Lu JJ, Liss AL, Hu C, Guo X, Wu Y, Zhang Y (2011). Radiation-induced cranial nerve palsy: a cross-sectional study of nasopharyngeal cancer patients after definitive radiotherapy. Int J Radiat Oncol Biol Phys.

[10] Matuschek C, Bölke E, Nawatny J, Hoffmann TK, Peiper M, Orth K, Gerber PA, Rusnak E, Lammering G, Budach W (2011). Bevacizumab as a treatment option for radiation-induced cerebral necrosis. Strahlenther Onkol.

[11] Pierzchala K, Pierzchala W, Rozek-Lesiak K (1994). Clinical and electrophysiological examination of brachial plexus in females after radiotherapy and surgical treatment for breast cancer. Neurol Neurochir Pol.

[12] Rubin DI, Schomberg PJ, Shepherd RF, Panneton JM (2001). Arteritis and brachial plexus neuropathy as delayed complications of radiation therapy. Mayo Clin Proc.

[13] Johansson S, Svensson H, Larsson LG, Denekamp J (2000). Brachial plexopathy after postoperative radiotherapy of breast cancer patients—a long-term follow-up. Acta Oncol.

[14] Spittle MF (1995). Brachial plexus neuropathy after radiotherapy for breast cancer. BMJ.

[15] Senkus-Konefka E, Jassem J (2006). Complications of breast-cancer radiotherapy. Clin Oncol.

[16] Bajrovic A, Rades D, Fehlauer F, Tribius S, Hoeller U, Rudat V, Jung H, Alberti W (2004). Is there a life-long risk of brachial plexopathy after radiotherapy of supraclavicular lymph nodes in breast cancer patients?. Radiother Oncol.

[17] Jaeckle KA (2010). Neurologic manifestations of neoplastic and radiation-induced plexopathies. Semin Neurol.

[18] Galecki J, Hicer-Grzenkowicz J, Grudzien-Kowalska M, Michalska T, Zalucki W (2006). Radiation-induced brachial plexopathy and hypofractionated regimens in adjuvant irradiation of patients with breast cancer—a review. Acta Oncol.

[19] Grégoire V, Levendag P, Ang KK, Bernier J, Braaksma M, Budach V, Chao C, Coche E, Cooper JS, Cosnard G, Eisbruch A, El-Sayed S, Emami B, Grau C, Hamoir M, Lee N, Maingon P, Muller K, Reychler H (2003). CT-based delineation of lymph node levels and related CTVs in the node-negative neck: DAHANCA, EORTC, GORTEC, NCIC, RTOG consensus guidelines. Radiother Oncol.

[20] Gregoire V, Eisbruch A, Hamoir M, Levendag P (2006). Proposal for the delineation of the nodal CTV in the node-positive and the post-operative neck. Radiother Oncol.

[21] Killer HE, Hess K (1990). Natural history of radiation-induced brachial plexopathy compared with surgically treated patients. J Neurol.

[22] Gosk J, Rutowski R, Urban M, Wiecek R, Rabczynski J (2007). Brachial plexus injuries after radiotherapy - analysis of 6 cases. Folia Neuropathol.

[23] Fathers E, Thrush D, Huson SM, Norman A (2002). Radiation-induced brachial plexopathy in women treated for carcinoma of the breast. Clin Rehabil.

[24] Dixon AK, Wheeler TK, Lomas DJ, Mackenzie R (1993). Computed tomography or magnetic resonance imaging for axillary symptoms following treatment of breast carcinoma? A randomized trial. Clin Radiol.

[25] Kneeland JB, Kellman GM, Middleton WD, Cates JD, Jesmanowicz A, Froncisz W, Hyde JS (1987). Diagnosis of diseases of the supraclavicular region by use of MR imaging. AJR Am J Roentgenol.

[26] Moore NR, Dixon AK, Wheeler TK, Freer CE, Hall LD, Sims C (1990). Axillary fibrosis or recurrent tumour. An MRI study in breast cancer. Clin Radiol.

[27] Wouter van Es H, Engelen AM, Witkamp TD, Ramos LM, Feldberg MA (1997). Radiation-induced brachial plexopathy: MR imaging. Skeletal Radiol.

[28] Iyer RB, Fenstermacher MJ, Libshitz HI (1996). MR imaging of the treated brachial plexus. AJR Am J Roentgenol.

[29] Hoeller U, Bonacker M, Bajrovic A, Alberti W, Adam G (2004). Radiation-induced plexopathy and fibrosis. Is magnetic resonance imaging the adequate diagnostic tool?. Strahlenther Onkol.

[30] Sham JS, Choy D, Wei WI (1990). Nasopharyngeal carcinoma: orderly neck node spread. Int J Radiat Oncol Biol Phys.

[31] Delanian S, Lefaix JL, Pradat PF (2012). Radiation-induced neuropathy in cancer survivors. Radiother Oncol.

[32] Tang Y, Rong X, Hu W, Li G, Yang X, Yang J, Xu P, Luo J (2014). Effect of edaravone on radiation-induced brain necrosis in patients with nasopharyngeal carcinoma after radiotherapy: a randomized controlled trial. J Neurooncol.

[33] Rong X, Tang Y, Chen M, Lu K, Peng Y (2012). Radiation-induced cranial neuropathy in patients with nasopharyngeal carcinoma. A follow-up study. Strahlenther Onkol.

[34] Tan T, Barry P, Reken S, Baker M, Guideline Development Group (2010). Pharmacological management of neuropathic pain in non-specialist settings: summary of NICE guidance. BMJ.

[35] Mocchetti I (2005). Exogenous gangliosides, neuronal plasticity and repair, and the neurotrophins. Cell Mol Life Sci.

[36] Gu B, Yang Z, Huang S, Xiao S, Zhang B, Yang L, Zhao J, Zhao Z, Shen J, Liu J (2014). Radiation-induced brachial plexus injury after radiotherapy for nasopharyngeal carcinoma. Jpn J Clin Oncol.

[37] Gosk J, Rutowski R, Reichert P, Rabczyński J (2007). Radiation-induced brachial plexus neuropathy - aetiopathogenesis, risk factors, differential diagnostics, symptoms and treatment. Folia Neuropathol.

[38] Behnke NK, Crosby SN, Stutz CM, Holt GE (2013). Periscapular amputation as treatment for brachial plexopathy secondary to recurrent breast carcinoma: a case series and review of the literature. Eur J Surg Oncol.

